# Pulsed Field Ablation for Atrial Fibrillation in Obesity: Reassessing Risk, Safety, and Success

**DOI:** 10.3390/jcm15155866

**Published:** 2026-07-27

**Authors:** Ridwan R. Waliyuddin, Dony Y. Hermanto, Sunu B. Raharjo, Dicky A. Hanafy, Yoga Yuniadi

**Affiliations:** 1 Department of Cardiology and Vascular Medicine, Faculty of Medicine, Universitas Indonesia, Jakarta 11420, Indonesia; dr.ridwan.rasyid@gmail.com (R.R.W.); donyugo@yahoo.com (D.Y.H.); sunu.b.raharjo@gmail.com (S.B.R.); drdhanafy@yahoo.de (D.A.H.); 2 National Cardiovascular Center Harapan Kita, Jakarta 11420, Indonesia

**Keywords:** atrial fibrillation, obesity, pulsed field ablation, radiofrequency ablation, cryoballoon ablation, epicardial adipose tissue

## Abstract

**Background:** Obesity is a major independent risk factor for atrial fibrillation (AF), contributing to adverse outcomes and complicating rhythm control. While lifestyle modification is recommended, catheter ablation remains central to management. Conventional thermal ablation techniques often yield suboptimal results in obese patients due to anatomical and biophysical challenges. Pulsed field ablation (PFA), a novel non-thermal modality, may overcome these limitations. **Methods:** A systematic search of PubMed, Scopus, and Embase identified observational cohort studies (2017–2026) evaluating PFA outcomes in obese AF patients or across BMI categories. Four cohort studies met the inclusion criteria. **Results:** Findings on AF recurrence were heterogeneous. Recurrence rates appeared lower with PFA compared to radiofrequency ablation (RFA), though differences were not statistically significant. In a PFA-only study, freedom from arrhythmia recurrence did not vary across BMI categories. Compared with cryoballoon ablation (CBA), one matched-cohort study demonstrated significantly higher one-year freedom from AF with PFA-PVI, whereas another reported no difference. Left atrial epicardial adipose tissue (LA EAT) emerged as the only independent predictor of recurrence in PFA patients, suggesting electrical field perturbation by fat tissue. Radiation exposure was lower with PFA than CBA, while fluoroscopy time and periprocedural complications were comparable across groups. **Conclusions:** Current observational evidence suggests that PFA may be a feasible and safe option for AF ablation in overweight and obese patients, with outcomes comparable to conventional ablation techniques. However, comparative efficacy and long-term safety remain uncertain. Obesity-related adipose hypertrophy, systemic inflammation, and atrial remodeling may alter electric field distribution, contributing to variable recurrence outcomes. Larger prospective and randomized trials are warranted to define the long-term role of PFA in this high-risk population.

## 1. Introduction

Atrial fibrillation (AF) and obesity are increasingly prevalent global cardiovascular health problems. Atrial fibrillation is the most common sustained arrhythmia found globally, with more than 59 million individuals living with AF in 2019 [1]. The prevalence of AF increases with advancing age, as aging is associated with a higher incidence of structural heart disease and metabolic conditions that alter electrical impulse conduction and autonomic nervous system activity, thereby promoting arrhythmogenesis [2].

Obesity is one of the major modifiable risk factors for AF that is also rising globally. Epidemiological data indicate that every 5-unit increment in body mass index (BMI) is independently associated with a 30% increase in the risk of AF incident [3]. Obesity promotes AF through structural remodeling, left atrial dilatation, and epicardial adipose tissue (EAT)-mediated inflammation and fibrosis, leading to conduction heterogeneity [4].

Conventional thermal ablation methods, namely radiofrequency ablation (RFA) and cryoballoon ablation (CBA), demonstrate compromised long-term efficacy in obese patients. As these modalities rely on thermal energy to create transmural lesions that target the arrhythmogenic substrate, the presence of adipose tissue may impede this process, making the procedure less effective. This limitation may increase the risk of pulmonary vein reconnection and arrhythmia recurrence [4].

Pulsed field ablation (PFA) is a novel non-thermal modality that induces cell apoptosis through irreversible electroporation. Compared with conventional thermal ablation techniques, PFA offers shorter procedure duration, tissue selectivity, and potentially lower complication rates. These characteristics may provide procedural advantages in obese patients, who are known to have higher procedural complexity, increased radiation exposure, and worse ablation outcomes [4,5]. However, evidence regarding the efficacy and safety of PFA specifically in overweight and obese AF patients remains limited.

Our systematic review aims to evaluate the outcomes of overweight and obese AF patients undergoing the PFA procedure compared with other conventional procedures and across different BMI classes.

## 2. Materials and Methods

This systematic review follows the updated Preferred Reporting Items for Systematic Reviews and Meta-Analyses (PRISMA) guidelines as revised in 2020 [6]. The two checklists were used as Supplementary Materials. (Supplementary Files S1 and S2). However, the study was not registered.

### 2.1. Literature Search

A comprehensive systematic literature search was conducted to evaluate studies in obese patients with AF undergoing catheter ablation (pulsed field, radiofrequency, and cryoballoon ablation). Studies focusing on the outcome compared or related to pulsed field ablation were included. The databases used to find literature were PubMed, Scopus, and Embase.

### 2.2. Search Strategy

The studies included in this review were limited to the publication years 2017–2026. The literature search was conducted on 1 May 2026, restricted to publications from 2017 onward, corresponding to the period when PFA first emerged as a clinically investigated ablation modality. Keywords used for this literature search were adjusted to the Medical Subject Headings (MeSHs) and indexing systems of each database, combined using Boolean operators. Database-specific search strings were applied as follows:

PubMed: (“Atrial Fibrillation”[MeSH Terms] OR “atrial fibrillation”[TIAB]) AND (“Obesity”[MeSH Terms] OR “obese”[TIAB] OR “overweight”[TIAB] OR “BMI”[TIAB]) AND (“Pulsed Field Ablation”[TIAB] OR “radiofrequency ablation”[MeSH Terms] OR “cryoablation”[MeSH Terms] OR “cryoballoon”[TIAB]) AND (“recurrence”[TIAB] OR “outcome”[TIAB] OR “safety”[TIAB] OR “complication”[TIAB]).

Scopus: TITLE-ABS-KEY (“atrial fibrillation” AND (“obesity” OR “obese” OR “overweight” OR “BMI”) AND (“pulsed field ablation” OR “radiofrequency ablation” OR “cryoablation” OR “cryoballoon”) AND (“recurrence” OR “outcome” OR “safety” OR “complication”)).

Embase: ‘atrial fibrillation’/exp AND (‘obesity’/exp OR ‘overweight’/exp OR ‘BMI’) AND (‘pulsed field ablation’ OR ‘radiofrequency ablation’/exp OR ‘cryoablation’/exp OR ‘cryoballoon’) AND (‘recurrence’ OR ‘outcome’ OR ‘safety’ OR ‘complication’).

### 2.3. Eligibility Criteria and Study Selection

Eligible studies had to fulfill the following criteria: (1) clinical trials or observational studies; (2) published in English between 2017 and 2026; (3) adult patients (>18 years) with AF undergoing PFA compared with RFA or CBA, or across different BMI categories; (4) patients classified as overweight (BMI 25–29.9 kg/m^2^) or obese (BMI ≥30 kg/m^2^) per WHO criteria, including mixed populations if outcomes were reported by BMI subgroup; and (5) studies reporting post-procedure outcome, including arrhythmia recurrence, radiation exposure, procedure duration, epicardial adipose tissue (EAT) parameters, or periprocedural complications. Additionally, we excluded studies with: (1) unavailable or inaccessible full-text articles; and (2) case reports, case series, review articles, editorials, and conference abstracts without sufficient data.

Study selection was conducted according to the PRISMA 2020 guidelines. Duplicate records were removed prior to title and abstract screening. Full-text articles were assessed for eligibility based on the predefined inclusion and exclusion criteria.

### 2.4. Data Extraction and Outcome Measures

The literature references from the database, including abstract and title, were imported in the format of an RIS file (.ris) into EndNoteX9. Title and abstract screening were performed independently by two authors (RRW, DYH), followed by an independent review of potentially eligible articles. Data extraction was performed independently by two authors (SBR, DAH) using a standardized extraction form. Disagreements were resolved through discussion and consensus, with arbitration by a senior author (YY) when necessary.

Extracted variables are as follows: study design, patient demographics, ablation modality and location, classification of body mass index (BMI), criteria for recurrence, follow-up duration, and clinical outcomes. The primary outcome of this review was atrial tachyarrhythmia recurrence following catheter ablation. Secondary outcomes included procedural duration, fluoroscopy time, EAT volume, and major and minor periprocedural complications. Due to the limited number of studies and significant heterogeneity in study design, patient populations, and outcome definitions, a formal meta-analysis was not feasible. Disparities in ablation modalities, BMI classifications, and recurrence criteria made pooled estimates inappropriate. Therefore, a systematic narrative synthesis was employed to evaluate the findings.

### 2.5. Quality Assessment

The quality of the included studies was assessed using the Newcastle–Ottawa Quality Assessment Scale (NOS) for cohort studies. This tool evaluates three dimensions that contribute to the overall quality score, including the selection of each cohort, the comparability of the cohorts, and outcome assessment. NOS is a star-based scoring system that awards a maximum of 4 stars in the selection domain, 2 stars in the comparability domain, and 3 stars in the outcome measurement domain. Studies with 7–9 stars are classified as high quality, 4–6 stars as moderate quality, and 0–3 as low quality [6].

## 3. Results

**Figure 1:**
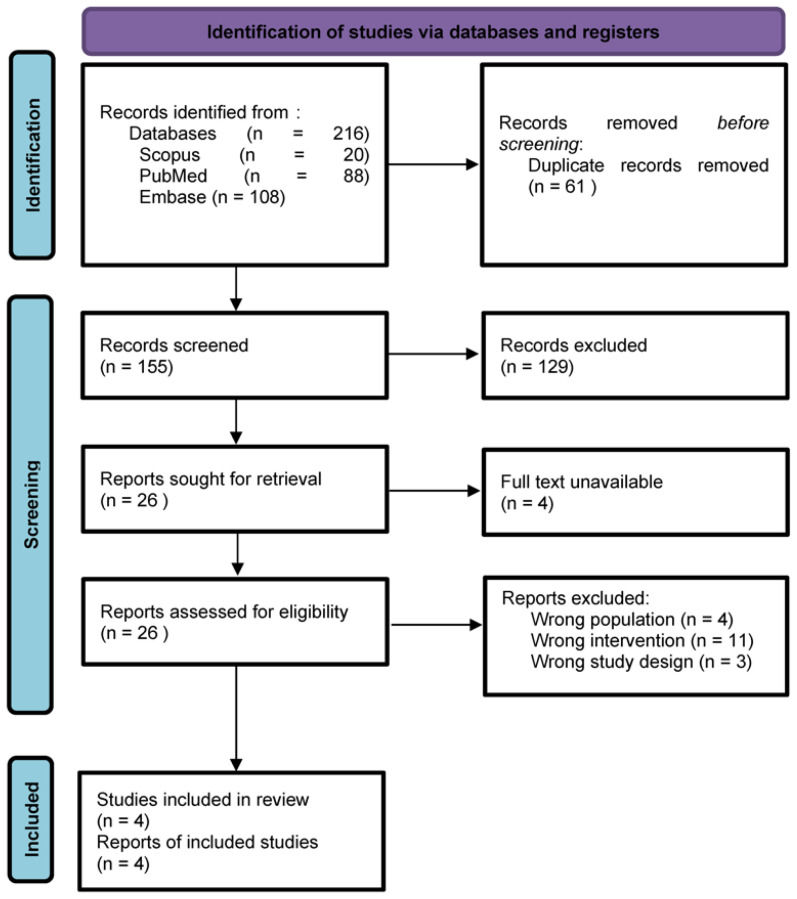
PRISMA flow diagram. The excluded studies and their specific reasons for exclusion were added in the Supplementary Table S1 [7,8,9,10,11,12,13,14,15,16,17,18,19,20,21,22,23,24,25,26,27,28].

A total of 216 records were identified through database searches, comprising 20 from Scopus, 88 from PubMed, and 108 from Embase. After removing 61 duplicates, 155 studies underwent title and abstract screening to assess eligibility. Of these, 129 were excluded due to inappropriate population, intervention, outcome measures, or study design. The remaining 26 articles proceeded to full-text review, resulting in the inclusion of four observational cohort studies in the final systematic analysis (Figure 1).

The journal matrix of included studies is presented in Table 1. Quality assessment of the included observational cohort studies was conducted using the Newcastle–Ottawa Scale (NOS), which shows that all included papers are classified as good quality (Table 2).

All patients included in this study are limited to paroxysmal and persistent AF types. Ha J et al. [30] performed ablation with a total of 99% receiving PVI, 40% with additional posterior wall ablation, and 3% with additional superior vena cava ablation. Englert et al. [29] performed PVI only, limited to paroxysmal AF patients, with additional cavotricuspid isthmus ablation with RF in cases of typical atrial flutter. The remaining two studies performed PVI alone without additional substrate modification.

### 3.1. Arrhythmia Recurrence

The study by Englert F. et al. [29] revealed that the recurrence of AF was observed less in patients who underwent PFA compared to the RF group (17.07% vs. 33.87%). However, this difference is not statistically significant (log-rank test; *p* = 0.077). The recurrence rate was counted at 1 year (excluding a 6-week blanking period), with an overall rate of 27.18%. A subgroup analysis of patients with heart-failure also showed no significant difference. Among patients without heart failure, recurrence rates remained higher in the RF group than the PFA group (29.7% vs. 15.6%; *p* = 0.04). Early recurrence was observed in two patients in the PFA group and one patient in the RF group, with 4.9% versus 1.5% (*p* = 0.664) requiring amiodarone after ablation due to AF recurrence.

Ha FJ. et al. [30] showed no significant differences in freedom from arrhythmia recurrence between groups based on the BMI category (68% in the nonobese group with median follow-up 5.9 months [IQR 2.6–13.7)] vs. 70% in the obese group with median follow-up 6.1 months [IQR 2.7–13.5]; *p* = 0.41 for difference). BMI, as a continuous variable, was not a significant predictor of arrhythmia recurrence (*p* = 0.86).

During the 1-year follow-up by Feickert et al. [31], arrhythmia recurrence was documented in 97 patients (29.5%) after a 90-day blanking period. Earlier recurrence was documented in the PFA group (CBA-PVI: 179 days, IQR 90–334; PFA-PVI: 143 days, IQR 90–334 days). No significant difference was observed in the 1-year recurrence rates between the two methods before propensity matching (CBA-PVI: 76 [31.3%]; PFA-PVI: 21 [24.4%]; *p* = 0.27). After propensity matching, freedom from AF, 1 year after catheter ablation, is significantly higher in patients undergoing PFA-PVI (n = 84) than in CBA-PVI (n = 84) (*p* = 0.02).

Jungen et al. [32] also observed no difference in the 1-year freedom from AF regarding the mode of ablation (PFA: 76% vs. CBA: 76%, HR: 1.37; 95% CI: 0.63–2.99; *p* = 0.42). For overweight (82%) and obese patients (67%), the 1-year AF-freedom was similar (HR: 0.61; 95% CI: 0.29–1.28; *p* = 0.19). Freedom from AF after 1 year is found to be higher in patients with paroxysmal AF (84%) compared to persistent AF (67%) (HR: 2.45; 95% CI: 1.1–5.4; *p* = 0.03). Using an eight-week blanking period, 75% (29 of 115) of patients were free from AF recurrence after 1 year. Neither the ablation mode (PFA: 74% vs. CBA: 76%, HR: 1.44; 95% CI: 0.66–3.11; *p* = 0.35) nor BMI (overweight: 82%, obese: 65%, HR: 0.61; 95% CI: 0.29–1.27; *p* = 0.19) showed a difference in 1-year AF recurrence. In patients with paroxysmal AF (84%), freedom from AF after 1 year was better compared to persistent AF (66%) (HR: 2.59; 95% CI: 1.18–5.69; *p* = 0.02) after an eight-week blanking period.

### 3.2. Procedural Outcomes

Besides the recurrence rate, procedure efficacy is associated with radiation exposure. Between groups stratified by BMI that underwent PFA, radiation exposure rose significantly with BMI for both air kerma (*p* < 0.001) and dose–area product (*p* < 0.001) despite similar fluoroscopy time between groups (median 15–17 min across groups; *p* = 0.15), as reported by Ha et al. [31].

The ablation method also affects radiation exposure. Jungen et al. [32] found that patients undergoing PFA-PVI had lower radiation exposure than patients undergoing CBA-PVI (PFA: 2196 cGy⋅cm^2^ [IQR: 1398–2973 cGy⋅cm^2^] vs. CBA: 3239 cGy⋅cm^2^ [IQR: 1288–5062], *p* = 0.009). Additionally, less contrast dye volume had to be applied in PFA- compared to CBA-PVI (PFA: 80 mL [IQR: 60–117 mL] vs. CBA: 130 mL [IQR: 95–200 mL], *p* = 0.001). The fluoroscopy time, as well as the rate of additional CTI ablations, did not differ between groups.

In patients undergoing CBA-PVI the radiation dose was higher in obese compared to overweight patients (CBA: obese: 4809 cGy⋅cm^2^ [IQR: 3253–5629 cGy⋅cm^2^] vs. overweight: 2258 cGy⋅cm^2^ [IQR: 988–3804 cGy⋅cm^2^], *p* = 0.039) and there was a trend for longer fluoroscopy time in obese compared to overweight patients (CBA: obese: 29 min. [IQR: 21–26 min.] vs. overweight: 23 min. [IQR: 17–30 min], *p* = 0.09). Multivariate logistic regression analysis revealed that obesity (OR: 5.58, 95% CI: 1.63–19.06; *p* = 0.006) and PVI by CBA (OR: 12.93, 95% CI: 3.51–47.68; *p* < 0.001) were associated with significantly higher radiation levels.

### 3.3. Role of Epicardial Adipose Tissue (EAT) in AF Recurrence

In the study by Englert F. et al. [29], LA EAT was the only significant predictor for recurrence, with an HR of 1.06 (95% CI 1.01–1.12; *p* = 0.022), with each additional milliliter of EAT conferring a 6.2% increased risk of arrhythmia recurrence. In the RFA group, increased LA EAT volume was not significantly associated with recurrent arrhythmia risk (HR 1.00; 95% CI 0.97–1.03; *p* = 0.846).

### 3.4. Safety and Complication Profile

In the study conducted by Englert et al. [29], there were no major complications, including pericardial effusion or stroke, in either group. Groin complications were observed in five patients (12.2%) of the PFA cohort and one patient (1.6%) of the RF cohort (*p* = 0.070).

Ha FJ. [8] showed no difference between BMI groups for the need for cardioversion (5–6%; *p* = 0.90), hospitalization related to arrhythmia (overall 2.8%; *p* = 2.9), or redo-ablation procedure (6.3%; *p* = 0.83).

Feickert et al. [31] reported no significant difference in periprocedural complications between the groups before or after propensity matching. Before matching, three cases of phrenic nerve palsy occurred in the CBA-PVI group, two pericardial effusion in each group (CBA-PVI: 1 [0.4%]; PFA-PVI: 1 [1.2%], both requiring pericardiocentesis), four pseudoaneurysms (CBA-PVI: 3 [1.2%]; PFA-PVI: 1 [1.2%]), and three arteriovenous fistulas (CBA-PVI: 2 [0.8%]; PFA-PVI: 1 [1.2%]).

Periprocedural adverse events were comparable between groups (PFA: 4% vs. CBA: 0%; *p* = 0.3). In the PFA group, two cases of pericardial tamponade were reported and successfully managed with pericardiocentesis without surgical intervention, while one patient developed an infected groin hematoma that prolonged hospitalization beyond 48 h, as reported by Jungen et al. [32].

## 4. Discussion

Obesity is the second leading modifiable risk factor for AF. Approximately 20% of overweight and obese individual will likely to develop AF. Obesity directly creates a proarrhythmic substrate through three pathways: chronic hemodynamic overload, local and systemic inflammation, and enhanced sympathetic nervous system [4].

In an obese state, the body’s total blood volume and cardiac output increase to meet the elevated metabolic demand, resulting in a chronic hemodynamic overload. This condition promotes left atrial enlargement due to an increase in left ventricular and left atrial pressures. Other comorbidities commonly found in obese patients, such as hypertension, insulin resistance, and diabetes, exacerbate hemodynamic load and create further cardiovascular strain [4].

Epicardial adipose tissue (EAT) is a visceral fat layer covering the atrial myocardium. EAT shares vascularization with the atria. Hypertrophic EAT changes its function into a highly active endocrine and paracrine organ, producing pro-inflammatory cytokines, pro-fibrotic adipokines, and reactive oxygen species. Additionally, epicardial adipocytes can infiltrate the myocardial layer and act as a mechanical barrier [4].

Obesity also affects neurohormonal changes through enhancing sympathetic nervous system activity and activation of the renin–angiotensin–aldosterone system, increasing vascular resistance, atrial stretch, and direct pro-fibrotic effects on the atrial myocardium. Collectively, these processes result in atrial structural remodeling characterized by left atrial enlargement, atrial inflammation, fibrosis, and fat-layer infiltration, which eventually leads to disruption of gap junction organization and slowed conduction. These changes create a heterogeneous atrial substrate that facilitates re-entry circuits and creates a sustained AF condition [4].

The obese condition may obscure the procedural process. Increased thickness of subcutaneous fat makes it difficult to identify anatomical landmarks and complicates femoral vein access, increasing the risk of vascular complications such as groin hematoma. Our study found that the rate of groin hematoma was higher in the PFA cohort compared to the RF cohort. However, this finding is more likely related to vascular access difficulty in generally obese patients rather than a modality-specific effect. Available evidence suggests that BMI-associated vascular events are more likely related to variations in vascular access technique, such as limited use of ultrasound guidance, rather than a modality-specific effect [33].

Subcutaneous fat may also affect procedural imaging and radiation exposure. Reduced image quality in patients with a higher body mass may lead to longer procedure times, increasing patients’ and operators’ exposure to X-ray fluoroscopy and its associated radiation doses [34]. Our study found that in the PFA-only procedure, higher BMI correlates with higher radiation exposure. Comparative studies across ablation modalities have reported inconsistent findings regarding radiation exposure. In a study comparing PFA to CBA, obese patients who underwent PFA had lower radiation exposure and less contrast dye volume. One study reports a lower cumulative radiation dose in the RF cohort compared to the PFA cohort. All studies report similar fluoroscopy times between PFA and other ablation modalities.

Additional imaging is needed in CBA due to the required monitoring of PV occlusion and phrenic nerve capture, which is needed in patients undergoing CBA-PVI but not in PFA-PVI, which might explain the similar fluoroscopy times with increased radiation dose [10]. This is consistent with findings from a larger meta-analysis in general AF populations, which shows no significant differences in fluoroscopy time or overall procedural duration between PFA and thermal ablation techniques. This variability across studies may be explained by differences in procedural workflow, patients’ characteristics, and operator experience [35].

The included studies demonstrated mixed findings regarding arrhythmia recurrence following PFA in obese AF patients. Englert et al. [29] observed lower recurrence rates in the PFA group compared with RF ablation, although the difference was not statistically significant. Jungen et al. [32] reported similar 1-year AF recurrence rates between PFA-PVI and CBA-PVI regardless of BMI category. Ha et al. [30] also demonstrated that BMI was not a significant predictor of arrhythmia recurrence after PFA. A significant difference in 1-year freedom from AF was found in the study by Feickert et al. [31] after propensity matching. Patients undergoing PFA-PVI show higher AF recurrence freedom compared with CBA-PVI. This shows that baseline characteristics of patients, such as age, heart’s structural and functional variation, AF type, and comorbidities, may affect recurrence besides different ablation techniques.

To provide a broader context for the outcomes identified in our systematic review, it is necessary to compare these findings with the established PFA literature involving the general AF population. Large-scale pivotal trials, including ADVENT and PULSED AF, have demonstrated that PFA is a highly effective modality with a favorable safety profile for both paroxysmal and persistent AF. These studies established its non-inferiority to thermal ablation while highlighting a significant reduction in collateral tissue injury risk [36,37]. Extensive real-world registries, such as MANIFEST-17K and EU-PORIA, further support these findings, reporting high acute success and a remarkably low incidence of major complications, such as phrenic nerve palsy or esophageal injury, across heterogeneous patient cohorts [33,38]. Furthermore, PFA outcomes in persistent AF have established a robust benchmark for rhythm control in patients with more complex arrhythmogenic substrates [5]. A recent meta-analysis by Mariani et al. comparing PFA to high power short-duration (HPSD) and very HPSD radiofrequency ablation demonstrated that PFA is associated with a significant reduction in AF recurrence in the overall population, while maintaining a comparable safety profile with no statistical differences in overall complications, stroke, or cardiac tamponade [39]. When benchmarking our obese cohort against these global standards, the fundamental safety and efficacy of PFA appear well-preserved. Nonetheless, the specific biophysical and anatomical challenges associated with obesity—including electric field perturbation by epicardial adipose tissue and advanced structural remodeling—likely account for the variable long-term recurrence rates observed in our analysis compared to the broader population.

Obesity remains associated with worse AF outcomes following catheter ablation due to more extensive atrial substrate remodeling beyond the pulmonary veins. Patients with obesity have a more extensive pathological substrate with a low-voltage area (LVA). LVA is a region where electrical signals measure < 0.5 mV, representing endocardial scar and atrial tissue with a structural defect. LVA is an independent predictor of ablation failure due to its nature in facilitating the re-entry circuit. LVA in obese patients often correlates with the regions of EAT deposition. This finding suggests a potential role for adjunctive substrate modification in this subgroup beyond just PVI ablation. Patients with obesity are also known to develop persistent AF, a condition that poorly responds to PVI ablation alone [4,40].

Most included studies in this review primarily performed PVI without extensive substrate modification, which may partially explain the comparable recurrence outcomes observed between ablation modalities. Lack of weight management post-procedure may also correlate with recurrence. Another study observed that even after PFA, each unit increase in BMI was associated with a 4.2% increase in the risk of AF recurrence [41].

One of the included studies showed that increased LA EAT volume was associated with a higher recurrence risk in patients undergoing PFA but not RFA. Although PFA has shown better tissue selectivity compared to thermal ablation, the tissue’s passive electrical properties and their relative spatial distribution also affect the resulting distribution of the electric field. In RFA and CBA, fat acts as a thermal insulator, limiting the creation of transmural lesions. However, other than thermal insulation, fat has lower electrical conductivity compared with healthy myocardium and fibrotic tissue. Mechanistically, it is hypothesized that fat may alter electric field distribution, resulting in higher electric field concentration within epicardial fat layers compared with the adjacent myocardium. When fat is distributed in isolated regions, computational models suggest that alterations in electric field strength may compromise the efficacy of PFA around arrhythmogenic foci adjacent to adipose tissue [42]. However, this remains a mechanistic speculation and requires further validation to be considered an established clinical association.

Additional evidence underscores the influence of adipose deposition on procedural durability, specifically highlighting how left atrial intramyocardial fat (inFAT) contributes to arrhythmia recurrence. Data from Landra et al. [43] revealed that inFAT was present at nearly 66.0% of pulmonary vein-left atrium (PV-LA) reconnection sites during redo procedures. Notably, segments characterized by electrical reconnection demonstrated significantly greater total inFAT volumes, encompassing both dense fat and fat-myocardial admixtures, when compared to segments that remained isolated. Given that adipose tissue exhibits markedly lower thermal and electrical conductivity than healthy myocardium, this three-dimensional architectural matrix may shield embedded cardiomyocytes from effective ablation. Such a barrier likely impedes the delivery of sufficient energy to ensure transmural lesion formation, potentially explaining the increased risk of recurrence observed in the obese population [43].

### Limitations

This systematic review has several limitations. First, the number of included studies is limited, with the study design only using observational and non-randomized cohorts. A formal meta-analysis was not conducted due to substantial heterogeneity across the four observational studies regarding comparative ablation modalities, BMI classification thresholds, and post-procedural blanking periods. Furthermore, variations in arrhythmia recurrence definitions across these studies precluded the calculation of reliable summary effect estimates. Consequently, a systematic narrative synthesis was employed to comprehensively evaluate and present the findings. Second, BMI classification, baseline criteria, follow-up duration, additional ablation strategies, and recurrence criteria differ among studies. Recurrence was defined as any atrial arrhythmia (AF, atrial flutter, or atrial tachycardia) in two studies [29,31], while Ha et al. [30] required symptomatic occurrence or documented arrhythmia ≥30 s, and Jungen et al. [32] required ECG- or Holter-confirmed episodes ≥30 s. Furthermore, the rhythm monitoring methodology was explicitly described only by Jungen et al. [32] using a 12-lead ECG combined with Holter monitoring, while the remaining studies did not specify their mode of arrhythmia detection, potentially underestimating asymptomatic recurrence. Third, most studies had small samples. Evidence evaluating obese patients undergoing PFA remains limited. The implementation of this novel technique is still affected by the operator’s unfamiliarity. Fourth, although all included studies achieved high NOS scores (8–9 stars), the NOS star score alone does not fully capture the nuances of bias inherent in observational research. All four studies were observational in design, with three retrospective and one prospective, with non-randomized treatment allocation, meaning patient assignment to PFA, RFA, or CBA was based on operator preference and institutional availability, introducing potential selection bias. Despite propensity score matching in Feickert et al. [9], residual confounding from unmeasured variables such as AF duration, comorbidity burden, and operator experience cannot be excluded. These methodological constraints limit the confidence in the conclusions drawn from this review.

## 5. Conclusions

Current observational evidence suggests that PFA may provide efficacy and safety comparable to conventional ablation techniques in obese patients, although definitive conclusions require randomized controlled trials. PFA appears feasible in overweight and obese patients, but its comparative efficacy and long-term safety remain uncertain. Complications are uncommon and largely related to body habitus; although a higher BMI increases radiation requirements, PFA generally reduces contrast and radiation compared with CBA.

The presence of epicardial adipose tissue may present a unique biophysical barrier; indeed, one cohort analysis identified left atrial EAT as a solitary independent factor predicting arrhythmia recurrence. From a mechanistic standpoint, it is hypothesized that the diminished electrical conductivity of fat relative to healthy myocardium might attenuate electric field intensity and compromise lesion transmurality, though such a clinical association necessitates further prospective validation. Since current evidence is based on small observational studies focused on pulmonary vein isolation, further randomized trials, adjunctive substrate modification, and weight management strategies are needed to optimize PFA outcomes.

## Figures and Tables

**Table 1:** Journal matrix of the included studies.

No.	Study Author	Study Type	Population	Modalities	Follow-Up and Blanking Period	Recurrence Criteria	Results
1.	Englert F, et al. (2025) [29].	Prospective cohort study	103 patients aged 18 years or older with BMI > 29 kg/m paroxysmal or short-term persistent AF who underwent first-time AF ablation (PFA n = 41, RFA n = 62).	PFA using the FARAPULSE endocardial ablation system compared with HPSD (High-Power, Short-Duration) RFA.	Median follow-up was 367 days, excluding a 6-week blanking period	Recurrence of symptomatic and asymptomatic any atrial arrhythmia occurred after the blanking period (3 months after ablation)	AF recurrence was lower in the PFA group compared to the RFA group (17.1% vs. 33.9%). Increased LA EAT volume was associated with higher recurrence risk in the PFA group (HR 1.06; 95% CI 1.01–1.12; *p* = 0.022), but not in the RFA group. Among patients without heart failure, recurrence remained higher in the RFA group (29.7% vs. 15.6%; *p* = 0.04). No major complications, including stroke or pericardial effusion, were reported. Groin complications occurred more frequently in the PFA group (12.2% vs. 1.6%; *p* = 0.070).
2.	Ha FJ, et al. (2025) [30].	Retrospective cohort study	537 patients were analysed in three groups according to BMI: <30 kg/m^2^ (nonobese), 30–34.9 kg/m^2^ (obese) and ≥35 kg/m^2^ (morbidly obese).	PFA using the Farapulse system	Median follow-up 5.9–7.4 months. No blanking period was applied.	Any symptomatic AF, atrial flutter, atrial tachycardia or documented arrhythmia lasting more than 30 s at any follow-up.	No significant difference in arrhythmia-free survival was observed across BMI groups (68% non-obese vs. 70% obese vs. 67% morbidly obese; *p* = 0.41), and BMI was not a significant predictor of recurrence. Rates of cardioversion, arrhythmia-related hospitalization, and redo ablation were comparable between groups. Radiation exposure increased significantly with BMI despite similar fluoroscopy times.
3.	Feickert et al. (2025) [31].	Retrospective cohort study	329 symptomatic patients with paroxysmal and persistent AF and a body mass index (BMI) >30 who underwent PFA or CBA at a single institution.	PFA or CBA PVI	Median follow-up of 1 year with 90-day blanking period applied	Any atrial arrhythmia occurred during 1-year follow-up post-procedure (atrial tachycardia, AF, atrial flutter).	Arrhythmia recurrence occurred in 29.5% of patients during 1-year follow-up. Before propensity matching, recurrence rates were comparable between PFA-PVI and CBA-PVI (24.4% vs. 31.3%; *p* = 0.27). After matching, freedom from AF at 1 year was significantly higher in the PFA-PVI group (*p* = 0.02).
4.	Jungen C, et al. (2024) [32].	Retrospective cohort study	115 symptomatic paroxysmal and persistent AF patients with a BMI ≥ 25 kg/m^2^. All patients were categorized into either over-weight (BMI 25–30 kg/m^2^) or obese (BMI > 30 kg/m^2^).	PFA- or CBA-PVI	Median follow-up 145 days. Blanking period of 3 months.	Any documented sustained AF on 12-lead ECG or any AF episode of 30 s or longer on Holter ECG.	No significant difference in 1-year AF recurrence was observed between PFA-PVI and CBA-PVI. Freedom from AF was higher in patients with paroxysmal AF than persistent AF. PFA-PVI was associated with lower radiation exposure and lower contrast dye use compared with CBA-PVI. Obesity and CBA-PVI were independently associated with higher radiation exposure. Periprocedural complications were comparable between groups, with two tamponades and one infected groin hematoma reported in the PFA group.

Note: AF = atrial fibrillation, PFA = pulse field ablation, RFA = radiofrequency ablation, CBA = cryo balloon ablation, LA = left atrial, EAT = epicardial adipose tissue, BMI = body mass index.

**Table 2:** Quality assessment of the included studies using the Newcastle–Ottawa Scale.

Study	Selection (0–4)				Comparability (0–2)		Outcome (0–3)			Total NOS
	Representativeness of exposed cohort	Selection of non-exposed cohort	Ascertainment of exposure	Outcome not present at start	Controls for most important confounder	Controls for additional confounder	Assessment of outcome	Length of follow-up	Adequacy of follow-up	
Englert F, et al. (2025) [29]	1	1	1	1	1	1	1	1	0	8/9
Ha FJ, et al. (2025) [30]	1	1	1	1	1	1	1	1	0	8/9
Feickert S, et al. (2025) [31]	1	1	1	1	1	1	1	1	1	9/9
Jungen C, et al. (2024) [32]	1	1	1	1	1	1	1	1	0	8/9

Note: high quality (7–9 score), moderate quality (4–6 score), poor quality (0–3 score).

## Data Availability

The study does not involve external datasets; all relevant data are presented in the manuscript.

## Supplementary Materials

The following supporting information can be downloaded at: https://www.mdpi.com/article/10.3390/jcm15155866/s1, Table S1: Specific reasons for article exclusion; Supplementary File S1: PRISMA_2020_checklist 1; Supplementary File S2: PRISMA_2020_abstract_checklist 2.

## Abbreviations

The following abbreviations are used in this manuscript:

AF	Atrial Fibrillation
PFA	Pulsed Field Ablation
BMI	Body Mass Index
RFA	Radiofrequency Ablation
CBA	Cryo-Balloon Ablation
PVI	Pulmonary Vein Isolation
LA	Left Atrial
EAT	Epicardial Adipose Tissue
PRISMA	Preferred Reporting Items for Systematic Reviews and Meta-Analyses
MeSH	Medical Subject Headings
RIS	Research Information System
NOS	Newcastle-Ottawa Quality Assessment Scale
HPSD	High-Power, Short-Duration
HR	Hazard Ratio
CI	Confidence Interval
IQR	Interquartile Range
LVA	Low Voltage Area

## References

[1] Linz D., Gawalko M., Betz K., Hendriks J.M., Lip G.Y., Vinter N., Guo Y., Johnsen S. (2024). Atrial fibrillation: Epidemiology, screening and digital health. Lancet Reg. Health—Eur..

[2] Iwasaki Yki Nishida K., Kato T., Nattel S. (2011). Atrial Fibrillation Pathophysiology. Circulation.

[3] Na J., Garapati S.S., Lador A. (2025). Obesity and Atrial Fibrillation: A Comprehensive Review. Methodist Debakey Cardiovasc. J..

[4] Cao Y., Gong A., Li Z., Li F., Hu X., Ren B., Li W., Zhou Y., Zeng R. (2026). A New Paradigm for Atrial Fibrillation Ablation in Obesity Focusing on Substrate Remodeling and Patient-Centered Outcomes. Rev. Cardiovasc. Med..

[5] Reddy V.Y., Anic A., Koruth J., Petru J., Funasako M., Minami K., Breskovic T., Sikiric I., Dukkipati S.R., Kawamura I. (2020). Pulsed Field Ablation in Patients With Persistent Atrial Fibrillation. J. Am. Coll. Cardiol..

[6] Page M.J., McKenzie J.E., Bossuyt P.M., Boutron I., Hoffmann T.C., Mulrow C.D., Shamseer L., Tetzlaff J.M., Akl E.A., Brennan S.E. (2021). The PRISMA 2020 statement: An updated guideline for reporting systematic reviews. BMJ.

[7] Choi S.-S., Kim K., Kim S.J., Hwang Y. (2026). Exploratory phase angle assessment after atrial fibrillation ablation in overweight patients: A pilot study. BMC Cardiovasc. Disord..

[8] Jiang Y., Zhou L., He L., Yang L., Liu F., Lu S., Li M., Jiang C., Tang R., Sang C. (2026). Differential Impact of Body Mass Index on Recurrence, Cardiac Remodelling, and Quality of Life After Atrial Fibrillation Ablation: Insights From the China-AF Registry. Diabetes Obes. Metab..

[9] Bar-Moshe A., Tsaban G., Dan I., Marai I., Michowitz Y., Glikson M., Luria D., Omelchenko A., Laish-Farkash A., Suleiman M. (2025). Does obesity affect atrial fibrillation ablation outcomes? Insights from a national atrial fibrillation catheter ablation registry: One-year follow-up. Heart Rhythm O_2_.

[10] Ling L.-C., Chang T.-Y., Lin C.-Y., Lin Y.-J., Hu Y.-F., Chung F.-P., Lo L.-W., Tuan T.-C., Chao T.-F., Liao J.-N. (2025). Impact of Obesity Phenotypes on Efficacy and Safety of Catheter Ablation for Patients with Paroxysmal Atrial Fibrillation. Acta Cardiol. Sin..

[11] Ma J., Wang C., He J., Zhang P., Pingzhen Y., Li X. (2025). H-type hypertension and U-shaped body mass index predict atrial fibrillation recurrence after catheter ablation: Insights from a 3-year follow-up. Heart Rhythm O_2_.

[12] Najafi S.A., Schaack D., Bordignon S., Urbani A., Kheir J., Garattini D., Steyer A., Tohoku S.H., Schmidt B., Chun K.R.J. (2025). Evaluation of effectiveness and outcomes of pulsed field ablation of atrial fibrillation in patients with obesity. Europace.

[13] Englert F., Bahlke F., Popa M.A., Lengauer S., Koops E., Tunsch A., Bicprendi P., Krafft H., Reiter T., Hessling G. (2025). Epicardial adipose tissue interference in atrial fibrillation (AF) ablation depending on energy sources: High power short duration vs. Pulsed Field energy. Europace.

[14] Ling L.-C., Chang T.-Y., Lin C.-Y., Lin Y.-J., Chang S.-L., Hu Y.-F., Chung F.-P., Lo L.-W., Tuan T.-C., Chao T.-F. (2025). PO-03-035 Impact of Obesity Phenotypes on Atrial Fibrillation Ablation Outcomes in an Asian Population. Heart Rhythm.

[15] Peng G., Thorne C., Varley A., Osorio J., Goyal S., Zei P. (2024). Abstract 4136804: Effect of Obesity on Arrhythmia Recurrence and Complications following Pulmonary Vein Isolation for Paroxysmal Atrial Fibrillation in the REAL-AF Registry. Circulation.

[16] Chamoun N., Chahine Y., Kassar A., Al Yasiri H., Hensley T., Haykal R., Boyle P.M., Akoum N. (2025). Reduction in Left Atrial Epicardial Adipose Tissue Following Catheter Ablation for Atrial Fibrillation. Circ. Arrhythmia Electrophysiol..

[17] Turagam M., Manifest pf Cooperative (2024). PO-06-157 Impact of body mass index on the outcomes of pulsed field ablation for atrial fibrillation: A MANIFEST-PF subanalysis. Heart Rhythm.

[18] Zhao H., Li X., Yu P., Liu M., Ma J., Wang J., Zhu W., Liu X. (2023). Association between weight loss and outcomes in patients undergoing atrial fibrillation ablation: A systematic review and dose–response meta-analysis. Nutr. Metab..

[19] Urbanek L., Bordignon S., Schaack D., Chen S., Plank K., Hirokami J., Schulte-Hahn B., Schmidt B., Chun K.R.J. (2023). Pulsed field versus cryoballoon ablation of atrial fibrillation: A comparison of efficacy and safety in a 400 patient cohort. Europace.

[20] Aleem S., Al-Sadawi M., Aslam F., Ijaz H., Cao K., Jacob R., Santore L., Almasry I., Fan R., Rashba E. (2022). Does body mass index affect atrial fibrillation ablation outcomes. Eur. Heart J..

[21] Blankoff I. (2022). Belgian Heart Rhythm Meeting 2022—Book of Abstracts. Acta Cardiol..

[22] Letsas K.P. (2020). Catheter ablation of atrial fibrillation in overweight and obese patients: Where do we stand?. Int. J. Cardiol..

[23] Şener Y.Z., Okşul M., Akkaya F. (2019). Predictors of recurrence after atrial fibrillation catheter ablation. Acta Cardiol..

[24] Monno K., Okumura Y., Saito Y., Aizawa Y., Nagashima K., Arai M., Watanabe R., Wakamatsu Y., Otsuka N., Yoda S. (2018). Effect of epicardial fat and metabolic syndrome on reverse atrial remodeling after ablation for atrial fibrillation. J. Arrhythmia.

[25] Zhou M., Wang H., Chen J., Zhao L. (2019). Epicardial adipose tissue and atrial fibrillation: Possible mechanisms, potential therapies, and future directions. Pacing Clin. Electrophysiol..

[26] Valli H., Tindale A., Butt H., Beattie C.J., Adasuriya G., Warraich M., Ahmad M., Banerjee A., Providencia R., Haldar S. (2024). Weight reduction interventions for the management of atrial fibrillation in overweight and obese people. Cochrane Database Syst. Rev..

[27] Farnir F., Luermans J., Manusama R., Uijl D.D., Chaldoupi S.M., Linz D. (2023). Focal point-by-point pulsed field ablation for the treatment of atrial arrhythmias in patients with challenging anatomy where radiofrequency ablation cannot be applied: A case series. Heart Case Rep..

[28] Urbanek L., Schmidt B., Bordignon S., Schaack D., Ebrahimi R., Tohoku S., Hirokami J., Efe T.H., Plank K., Schulte-Hahn B. (2024). Cryoablation of atrial fibrillation in “very severe” obese patients (BMI ≥ 40): Indications, feasibility, procedural safety and efficacy, and clinical outcome (the ICE-Obese Extreme). J. Cardiovasc. Electrophysiol..

[29] Englert F., Obermeyer T., Bahlke F., Popa M., Krafft H., Martinez A.T., Syväri J., Tydecks M., Dischel D., Koops E. (2025). Epicardial adipose tissue and ablation outcomes in obese patients with paroxysmal atrial fibrillation: A comparison of pulsed field and radiofrequency ablation. Heart Rhythm O_2_.

[30] Ha F.J., Prinsloo D., Xu E., Han H.C., Nerlekar N., Brown A.J., Kotschet E. (2026). Pulsed Field Ablation for Atrial Fibrillation in Patients With Obesity: Procedural and Clinical Outcomes. Pacing Clin. Electrophysiol..

[31] Feickert S., Wagner S., Biernath K., Ince H., Ortak J., Boehmer A.A., D’aNcona G., Ewertsen N.C. (2025). One-year clinical and safety outcome of obese patients undergoing pulmonary vein isolation for atrial fibrillation with pulsed field ablation or cryoballoon ablation: A propensity-matched analysis. Heart Rhythm.

[32] Jungen C., Rattka M., Bohnen J., Mavrakis E., Vlachopoulou D., Dorna S., Rudolph I., Kohn C., Dobrev D., Rassaf T. (2024). Impact of overweight and obesity on radiation dose and outcome in patients undergoing pulmonary vein isolation by cryoballoon and pulsed field ablation. IJC Heart Vasc..

[33] Ekanem E., Neuzil P., Reichlin T., Kautzner J., van der Voort P., Jais P., Chierchia G.-B., Bulava A., Blaauw Y., Skala T. (2024). Safety of pulsed field ablation in more than 17,000 patients with atrial fibrillation in the MANIFEST-17K study. Nat. Med..

[34] Ector J., Dragusin O., Adriaenssens B., Huybrechts W., Willems R., Ector H., Heidbüchel H. (2007). Obesity Is a Major Determinant of Radiation Dose in Patients Undergoing Pulmonary Vein Isolation for Atrial Fibrillation. J. Am. Coll. Cardiol..

[35] Aldaas O.M., Malladi C., Aldaas A.M., Han F.T., Hoffmayer K.S., Krummen D., Ho G., Raissi F., Birgersdotter-Green U., Feld G.K. (2023). Safety and acute efficacy of catheter ablation for atrial fibrillation with pulsed field ablation vs thermal energy ablation: A meta-analysis of single proportions. Heart Rhythm O_2_.

[36] Reddy V.Y., Gerstenfeld E.P., Natale A., Whang W., Cuoco F.A., Patel C., Mountantonakis S.E., Gibson D.N., Harding J.D., Ellis C.R. (2023). Pulsed Field or Conventional Thermal Ablation for Paroxysmal Atrial Fibrillation. N. Engl. J. Med..

[37] Verma A., Haines D.E., Boersma L.V., Sood N., Natale A., Marchlinski F.E., Calkins H., Sanders P., Packer D.L., Kuck K.-H. (2023). Pulsed Field Ablation for the Treatment of Atrial Fibrillation: PULSED AF Pivotal Trial. Circulation.

[38] Kueffer T., Bordignon S., Neven K., Blaauw Y., Hansen J., Adelino R., Ouss A., Füting A., Roten L., Mulder B.A. (2024). Durability of Pulmonary Vein Isolation Using Pulsed-Field Ablation. JACC Clin. Electrophysiol..

[39] Mariani M.V., Matteucci A., Pierucci N., Palombi M., Compagnucci P., Bruti R., Cipollone P., Vinciullo S., Trivigno S., Piro A. (2025). Pentaspline Pulsed Field Ablation Versus High-Power Short-Duration/Very High-Power Short-Duration Radiofrequency Ablation in Atrial Fibrillation: A Meta-Analysis. J. Cardiovasc. Electrophysiol..

[40] Liu H.T., Yang C.H., Lee H.L., Chang P.C., Wo H.T., Wen M.S., Wang C.-C., Yeh S.-J., Chou C.-C. (2021). Clinical Outcomes of low-voltage area-guided left atrial linear ablation for non-paroxysmal atrial fibrillation patients. PLoS ONE.

[41] Telesca A., Bordignon S., Neven K., Reichlin T., Blaauw Y., Hansen J., Boveda S., Ouss A., Mulder B.A., Ruwald M.H. (2024). Procedural and outcome impact of body mass index on pulsed field ablation of atrial fibrillation: Insights from the EU-PORIA Registry. Europace.

[42] Pérez J.J., González-Suárez A. (2023). How intramyocardial fat can alter the electric field distribution during Pulsed Field Ablation (PFA): Qualitative findings from computer modeling. PLoS ONE.

[43] Landra F., Saglietto A., Falasconi G., Penela D., Soto-Iglesias D., Curti E., Tonello B., Teresi L., Turturiello D., Franco-Ocaña P. (2025). Left atrial intramyocardial fat at pulmonary vein reconnection sites during atrial fibrillation redo ablation. Europace.

