# Supplementary material for: Pulsed Field Ablation for Atrial Fibrillation in Obesity: Reassessing Risk, Safety, and Success
Source: J Clin Med. 2026 Jul 27;15(15):5866. doi: 10.3390/jcm15155866 (PMC13467571; doi:10.3390/jcm15155866)
Supplement: Supplementary file 1 [file jcm-15-05866-s001.zip › jcm-4382098-supplementary-XML.pdf]

Supplementary Table

Table S1. Specific Reasons for article exclusion

No.	Title	Author, year	Reason for exclusion
1.	Exploratory phase angle assessment after atrial fibrillation ablation in overweight patients: a pilot study	Choi S, et al (2026) [7]	Wrong intervention<br>Focused on body composition outcomes rather than ablation modality comparison.
2.	Differential impact of body mass index on recurrence, cardiac remodelling, and quality of life after atrial fibrillation ablation: insights from the China-AF registry	Jiang Y, et al (2026) [8]	Wrong intervention<br>Did not evaluate PFA as intervention of interest or comparing different ablation modalities outcome.
3.	Does obesity affect atrial fibrillation ablation outcomes? Insights from a national atrial fibrillation catheter ablation registry: one-year follow-up	Bar-Moshe A, et al (2026) [9]	Wrong intervention<br>Did not evaluate PFA as intervention of interest or comparing different ablation modalities outcome.
4.	Impact of obesity phenotypes on efficacy and safety of catheter ablation for patients with paroxysmal atrial fibrillation	Ling L, et al (2025) [10]	Wrong intervention<br>Did not evaluate PFA as intervention of interest.
5.	H-type hypertension and u-shaped body mass index predict atrial fibrillation	Ma J, et al (2025) [11]	Wrong intervention

	recurrence after catheter ablation: insights from a 3-year follow-up		Only assessing recurrence post-RFA did not evaluate PFA as intervention of interest or comparing different ablation modalities outcome.
6.	Evaluation of effectiveness and outcomes of pulsed field ablation of atrial fibrillation in patients with obesity	Aminolsharieh NS, et al (2025) [12]	Full text unavailable
7.	Po-03-002 epicardial adipose tissue interference in atrial fibrillation (af) ablation depending on energy sources: high power short duration vs. Pulsed field energy	Englert F, et al (2025) [13]	Full text unavailable
8.	Po-03-035 impact of obesity phenotypes on atrial fibrillation ablation outcomes in an Asian population	Ling L, et al (2025) [14]	Wrong intervention<br>Did not evaluate PFA as intervention of interest or comparing different ablation modalities outcome.
9.	Effect of obesity on arrhythmia recurrence and complications following pulmonary vein isolation for paroxysmal atrial fibrillation in the Real-AF registry	Peng G, et al (2024) [15]	Wrong intervention<br>Did not evaluate PFA as intervention of interest or comparing different ablation modalities outcome.

10.	Reduction in left atrial epicardial adipose tissue following catheter ablation for atrial fibrillation	Chamoun N, et al (2025) [16]	Wrong intervention<br>Evaluated post-ablation eat remodeling rather than comparing ablation modalities.
11.	Po-06-157 impact of body mass index on the outcomes of pulsed field ablation for atrial fibrillation: a manifest-pf subanalysis	Turagam MK, et al (2024) [17]	Full text unavailable
12.	Association between weight loss and outcomes in patients undergoing atrial fibrillation ablation: a systematic review and dose–response meta-analysis	Zhao H, et al (2023) [18]	Wrong study design<br>Systematic review and meta-analysis.
13.	Pulsed field versus cryoballoon ablation of atrial fibrillation: a comparison of efficacy and safety in a 400-patient cohort	Urbanek L, et al (2023) [19]	Wrong population<br>Included a general AF population with no analysis according to BMI status.
14.	Does body mass index affect atrial fibrillation ablation outcomes	Aleem S, et al (2022) [20]	Wrong intervention<br>Did not evaluate pfa as intervention of interest or comparing different ablation modalities outcome.
15.	Body mass index as a predictor of atrial fibrillation recurrence after catheter	Berners Y, et al (2022) [21]	Full text unavailable

	ablation: experience from a high- volume Belgian tertiary center		
16.	Catheter ablation of atrial fibrillation in overweight and obese patients: where do we stand?	Letsas KP, et al (2020) [22]	Wrong study design<br>Narrative editorial article
17.	Predictors of recurrence after atrial fibrillation catheter ablation	Sener YZ, et al (2020) [23]	Wrong population, wrong study design. Included a general AF population. Did not report original clinical outcomes in an eligible AF cohort.
18.	Effect of epicardial fat and metabolic syndrome on reverse atrial remodeling after ablation for atrial fibrillation	Monno K, et al (2018) [24]	Wrong intervention<br>Focused on eat/metabolic syndrome rather than comparison of different ablation modalities outcome.
19.	Epicardial adipose tissue and atrial fibrillation: possible mechanisms, potential therapies, and future directions	Zhou M, et al (2020) [25]	Wrong study design<br>Narrative review article.
20.	Weight reduction interventions for the management of atrial fibrillation in overweight and obese people	Valli H, et al (2024) [26]	Wrong intervention did not evaluate the outcome of ablation.
21.	Focal point-by-point pulsed field ablation for the treatment of atrial arrhythmias in	Farnir F et al (2024) [27]	Wrong population

	patients with challenging anatomy where radiofrequency ablation cannot be applied: a case series		Assessed patients with difficult anatomic approach other than BMI issue.
22.	Catheter ablation of atrial fibrillation in "very severe" obese patients (BMI≥ 40): insights from "the ice-obese extreme" study	Chen S, et al (2024) [28]	Wrong study design and wrong population<br>Reply article rather than original clinical study, only includes very severe obese patients.
